# Expression Variants of the Lipogenic *AGPAT6* Gene Affect Diverse Milk Composition Phenotypes in *Bos taurus*

**DOI:** 10.1371/journal.pone.0085757

**Published:** 2014-01-21

**Authors:** Mathew D. Littlejohn, Kathryn Tiplady, Thomas Lopdell, Tania A. Law, Andrew Scott, Chad Harland, Ric Sherlock, Kristen Henty, Vlad Obolonkin, Klaus Lehnert, Alistair MacGibbon, Richard J. Spelman, Stephen R. Davis, Russell G. Snell

**Affiliations:** 1 Research & Development, Livestock Improvement Corporation, Hamilton, New Zealand; 2 School of Biological Sciences, University of Auckland, Auckland, New Zealand; 3 Nutrition and Bioactives, Fonterra Research Centre, Palmerston North, New Zealand; University of Queensland, Australia

## Abstract

Milk is composed of a complex mixture of lipids, proteins, carbohydrates and various vitamins and minerals as a source of nutrition for young mammals. The composition of milk varies between individuals, with lipid composition in particular being highly heritable. Recent reports have highlighted a region of bovine chromosome 27 harbouring variants affecting milk fat percentage and fatty acid content. We aimed to further investigate this locus in two independent cattle populations, consisting of a Holstein-Friesian x Jersey crossbreed pedigree of 711 F2 cows, and a collection of 32,530 mixed ancestry *Bos taurus* cows. Bayesian genome-wide association mapping using markers imputed from the Illumina BovineHD chip revealed a large quantitative trait locus (QTL) for milk fat percentage on chromosome 27, present in both populations. We also investigated a range of other milk composition phenotypes, and report additional associations at this locus for fat yield, protein percentage and yield, lactose percentage and yield, milk volume, and the proportions of numerous milk fatty acids. We then used mammary RNA sequence data from 212 lactating cows to assess the transcript abundance of genes located in the milk fat percentage QTL interval. This analysis revealed a strong eQTL for *AGPAT6,* demonstrating that high milk fat percentage genotype is also additively associated with increased expression of the *AGPAT6* gene. Finally, we used whole genome sequence data from six F1 sires to target a panel of novel *AGPAT6* locus variants for genotyping in the F2 crossbreed population. Association analysis of 58 of these variants revealed highly significant association for polymorphisms mapping to the 5′UTR exons and intron 1 of *AGPAT6*. Taken together, these data suggest that variants affecting the expression of *AGPAT6* are causally involved in differential milk fat synthesis, with pleiotropic consequences for a diverse range of other milk components.

## Introduction

The lactating mammary gland is a sophisticated secretory organ, producing a complex mixture of lipids, proteins, carbohydrates and various vitamins and minerals as a source of nutrition for the developing young. The relative proportions of these milk components vary widely both between and within species [1], with some of this variability attributable to genetics. In *Bos taurus,* large scale genetic studies have led to the identification of numerous genomic regions affecting the abundance of major milk components [2]–[5]. Although quantitative trait loci (QTL) for differential milk composition have been detected on most bovine autosomes, few of the causative genes underlying these signals have been identified. Of those genes with confirmed effects, the most studied is diacylglycerol acyltransferase 1 (*DGAT1*). Variant forms of *DGAT1* have been shown to have major effects on milk fat percentage, yield, and composition, protein percentage and yield, and milk volume [6], [7]. The effects of *DGAT1* on milk fat composition reflect its role as a key acyltransferase of the mammary triglyceride synthesis pathway, responsible for catalysing diacylglycerol to triacylgycerol [8].

Several recent genome-wide association studies (GWAS) have highlighted a region of bovine chromosome 27 affecting the lipid composition of milk [9]–[11]. Although the causative gene underlying these QTL has not been functionally demonstrated, *AGPAT6* has been proposed as a candidate for these effects [9]–[11]. The *AGPAT6* gene represents an excellent positional candidate in this regard since, like *DGAT1*, *AGPAT6* plays pivotal roles in milk fat synthesis. Triglyceride synthesis occurs through the stepwise addition of fatty acyl groups to glycerol-3-phosphate, with DGATs catalysing the last step in this chain, and 1-acylglycerol-3-phosphate acyltransferases (AGPATs) an intermediary step [12]. In the bovine mammary gland, *AGPAT6* appears to be the most abundant AGPAT isoform, with *AGPAT6* expression strongly upregulated during lactation [13]. Knockout of the *AGPAT6* gene in mice also produces animals with defects in lactation, where milk from double knockout animals is depleted in diacylglycerols and triacylglycerols by approximately 90% [14].

In the current study, we aimed to further investigate the chromosome 27 milk fat percentage locus. Using markers imputed from the Illumina BovineHD chip, association analysis was conducted to assess variant effects on milk lipid content and a range of other milk production and composition phenotypes. We also report use of RNA sequencing (RNAseq) and quantitative PCR (qPCR) analysis in lactating mammary tissue to conduct expression QTL mapping of *AGPAT6* and other genes in the milk fat percentage QTL interval. Finally, we used whole genome sequence data to investigate a range of novel, candidate causative *AGPAT6* variants for association with milk fat percentage.

## Results

### Genome-wide Association Analysis Identifies a Chromosome 27 QTL for Milk Fat Percentage in Two Independent Cattle Populations

**Figure 1:**
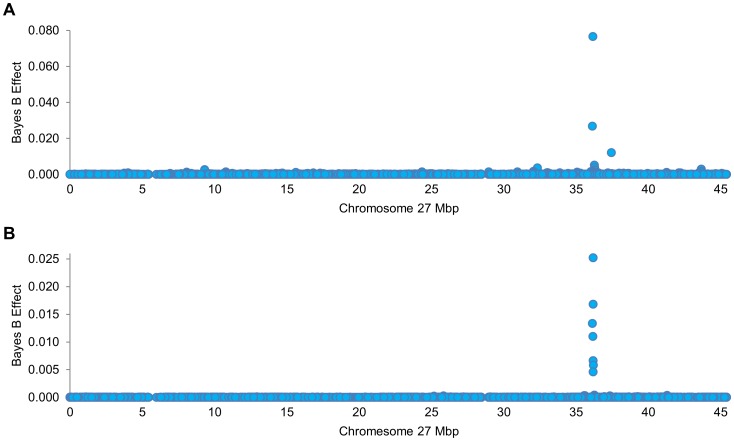
Milk fat percentage QTL on chromosome 27. Chromosome 27 Manhattan plots showing milk fat percentage QTL for the mixed ancestry (n = 32,530; Figure 1A) and Friesian-Jersey crossbreed (n = 711; Figure 1B) populations. The X-axis shows Mbp position on chromosome 27 according to the UMD3.1 *Bos taurus* genome build, the Y-axis shows the absolute value of the allele substitution effect on milk fat percentage as estimated using Bayes B models.

Bayes B association mapping using 653,725 genome-wide SNP markers in 32,530 MA cows revealed a strong QTL for milk fat percentage on chromosome 27, with the largest effect estimated for the ARS-BFGL-NGS-57448 SNP chr27 g.36155097C>T on the UMD3.1 genome build (Figure 1A; Table S1). Relative to gene annotations derived from mammary RNA sequence data, this SNP maps to intron 6 of the GINS4 gene, ∼29 kbp downstream of *GOLGA7*, and 43 kbp upstream of *AGPAT6*. When estimating the combined effect of markers partitioned into 1 Mbp windows throughout the genome, this signal was the second largest milk fat percentage QTL genome-wide, with the largest effect estimated for the well-studied *DGAT1* locus on chromosome 14 (Table S2). In MA animals, 54.4% of the variance in milk fat percentage was explained by all fitted SNPs, with the chromosome 27 36–37 Mbp window of markers accounting for 1.5% of the genetic variance. Association analysis using the same set of high density SNP markers used in the analysis of MA cows was conducted in a crossbreed population of 711 F2 animals. This analysis similarly revealed a large QTL for milk fat percentage on chromosome 27, with the largest effect estimated for the BovineHD2700010331 SNP chr27 g.36175805C>T (Figure 1B; Table S3). This SNP maps ∼50 kbp downstream of *GOLGA7*, ∼17 kbp downstream of *GINS4*, and ∼22 kbp upstream of *AGPAT6*. Consistent with the findings in the MA population, the chromosome 27 QTL was the second largest milk fat percentage QTL in the genome using window analysis, with the largest effect corresponding to the *DGAT1* locus (Table S4). In FJX animals, 18.4% of the variance in milk fat percentage was explained by all fitted SNPs, with the chromosome 27 36–37 Mbp window of markers accounting for 3.4% of the genetic variance.

### Chromosome 27 Milk Fat Percentage Locus Variants Associate with Diverse Milk Composition Phenotypes

Given the effects of chromosome 27 variants on milk fat percentage indicated by Bayes B analysis, we investigated a wide range of other phenotypes to test for pleiotropic effects on milk composition at this locus. Point-wise associations were analysed using the ARS-BFGL-NGS-57448 SNP, since this variant was highly associated with milk fat percentage in both the MA and FJX populations (Table S1, Table S3), and data representing this variant comprised real (versus imputed) genotypes. These models differed from the Bayes B models used for genome-wide association in that they fitted a single SNP at a time, and accounted for pedigree relationships by fitting a pedigree-based relationship matrix. In the MA population, the ARS-BFGL-NGS-57448 SNP was significantly associated with all milk composition traits tested (Table 1). Highly significant associations were found for total milk volume (5.17×10^−11^), total protein yield (P = 8.38×10^−9^), and total lactose yield and percentage (P = 3.70×10^−8^ and P = 1.49×10^−29^ respectively). The most significant association was for milk fat percentage (P = 5.31×10^−86^), with this SNP accounting for 1.6% of the genetic variance, and 1.3% of the phenotypic variance in MA animals. The allele frequency of the high-fat percentage ‘C’ allele of the ARS-BFGL-NGS-57448 SNP was 0.60 in the MA population. When animals in this population were divided into ‘pure’ Holstein-Friesian and Jersey subgroups (at least 13/16ths ancestry), the frequencies of this allele were 0.71 and 0.47 respectively. In FJX animals, the most significant association was also for milk fat percentage (8.90×10^−10^), with the ARS-BFGL-NGS-57448 SNP accounting for 7.8% of the genetic variance, and 5.7% of the phenotypic variance. Significant effects were also demonstrated for milk fat yield (P = 0.02), and lactose yield and percentage (P = 0.01 and P = 9.89×10^−6^ respectively; Table 1). In FJX animals, the ARS-BFGL-NGS-57448 SNP was also significantly associated with the proportions of a range of milk fatty acids (Table 1; Table S5). The high milk fat percentage allele corresponded to increased proportions of saturated fatty acids (P = 4.63×10^−4^) and C16 fatty acids (P = 5.22×10^−6^). Reciprocally, the same allele was associated with decreases in unsaturated fatty acids (P = 4.60×10^−4^), and reductions in omega-3 and omega-6 fatty acids (P = 1.25×10^−3^ and P = 1.91×10^−4^), cis-monoenoic fatty acids (P = 2.40×10^−4^), poly-unsaturated fatty acids (P = 2.88×10^−3^), and fatty acids with a chain-length longer than C16 (P = 0.01; Table 1). Association statistics for individual fatty acids within each of the above categories are detailed in Table S5. The allele frequency of the high-fat percentage ‘C’ allele of the ARS-BFGL-NGS-57448 SNP was 0.54 in the FJX population.

**Table 1:** Chromosome 27 locus effects on milk composition phenotypes.

			Parameter-adjusted means				
	Animal N	Parameter Estimate	TT	TC	CC	Pheno variance	p-value
**MA Animals - Gross Milk Composition**							
Milk fat %	32530	0.0991 (±0.0050)	4.814	4.913	5.012	1.269	5.31×10^−86^
Milk fat yield	32528	0.0042 (±0.0013)	0.711	0.715	0.719	0.034	1.33×10^−3^
Milk protein %	32505	0.0080 (±0.0024)	3.827	3.835	3.843	0.039	7.17×10^−4^
Milk protein yield	32530	−0.0058 (±0.0010)	0.573	0.567	0.561	0.110	8.38×10^−9^
Milk lactose %	19519	−0.0180 (±0.0016)	5.173	5.155	5.137	0.727	1.49×10^−29^
Milk lactose yield	19635	−0.0104 (±0.0018)	0.712	0.701	0.691	0.167	3.70×10^−8^
Milk volume	32528	−0.1812 (±0.0276)	15.116	14.935	14.753	0.143	5.17×10^−11^
**FJX Animals - Gross Milk Composition**							
Milk fat %	711	0.1631 (±0.0262)	5.162	5.325	5.488	5.733	8.90×10^−10^
Milk fat yield	711	0.0110 (±0.0047)	0.649	0.660	0.671	0.939	0.020
Milk protein %	711	0.0248 (±0.0136)	3.928	3.952	3.977	0.481	0.070
Milk protein yield	711	−0.0047 (±0.0035)	0.498	0.493	0.488	0.315	0.179
Milk lactose %	711	−0.0295 (±0.0066)	4.944	4.914	4.885	3.314	9.89×10^−6^
Milk lactose yield	711	−0.0128 (±0.0052)	0.626	0.614	0.601	1.071	0.013
Milk volume	711	−0.1759 (±0.0995)	12.892	12.716	12.540	0.546	0.078
**FJX Animals – Milk Fatty Acid Composition**							
C4–C15	671	−0.1465 (±0.1142)	30.468	30.321	30.175	0.082	0.200
C16	671	0.5778 (±0.1258)	28.473	29.051	29.628	1.377	5.22×10^−6^
C17–C24	671	−0.4309 (±0.1758)	41.008	40.577	40.146	0.585	0.015
Omega-3	671	−0.0194 (±0.0060)	1.141	1.122	1.102	0.486	1.25×10^−3^
Omega-6	671	−0.0218 (±0.0058)	1.308	1.286	1.264	1.363	1.91×10^−4^
Saturated	671	0.4412 (±0.1253)	69.862	70.303	70.744	1.248	4.63×10^−4^
Unsaturated	671	−0.4413 (±0.1253)	30.138	29.697	29.255	1.245	4.60×10^−4^
Poly-unsaturated	671	−0.0670 (±0.0224)	4.267	4.200	4.133	0.971	2.88×10^−3^
Branched	671	−0.0162 (±0.0102)	2.079	2.063	2.047	0.247	0.113
Cis-monoenoic	671	−0.3792 (±0.1027)	21.122	20.743	20.364	1.325	2.40×10^−4^
Trans-11-monoenoic	671	0.0029 (±0.0384)	4.760	4.763	4.766	0.001	0.939

Genetic associations with the ARS-BFGL-NGS-57448 are shown for the mixed ancestry (MA) and Friesian-Jersey crossbreed (FJX) populations. The number of individuals used in each analysis is indicated in the ‘Animal N’ column. Parameter estimates are given with standard errors in units of kilograms for yield traits, litres for milk volume, and g/100 g of fatty acids for milk fatty acids. Parameter adjusted means are indicated in the same units. The percentage of the total phenotypic variance explained by the ARS-BFGL-NGS-57448 SNP for each trait is indicated in the ‘Pheno variance’ column. P-values of genetic association are indicated in the right-most column.

### Milk Fat Percentage QTL Genotype Associates with *AGPAT6* Transcript Abundance in the Lactating Mammary Gland

**Figure 2:**
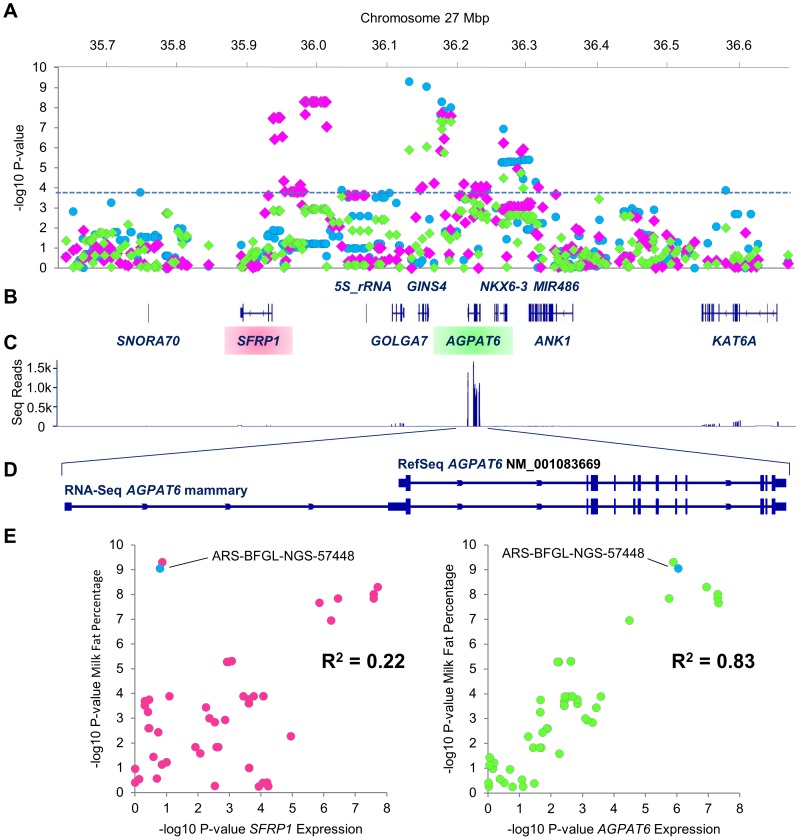
Expression QTL analysis at the milk fat percentage locus. Layered Manhattan plots for the milk fat percentage QTL in Friesian-Jersey crossbreed animals (blue dots), the *SFRP1* and *AGPAT6* eQTL in RNA-sequenced animals (pink and green triangles respectively; Figure 2A). The X-axis shows Mbp position on chromosome 27, the Y-axis shows –log_10_ p-values of marker association. The dashed blue line indicates nominal marker significance. Figure 2B indicates the ten genes mapping to the 1 Mbp interval, Figure 2C shows mean RNA sequence read depth across the interval. Figure 2D indicates alternative *AGPAT6* transcript structures according to the RefSeq entry NM_001083669.1, and the structure determined empirically from mammary RNA sequence data. Figure 2E shows correlation plots based on association data from individual markers for the milk fat percentage QTL and *SFRP1* eQTL (pink dots) and *AGPAT6* eQTL (green dots). The X-axes indicate the –log_10_ p-values of marker association for expression phenotypes, the Y-axes indicate the –log_10_ p-values for milk fat percentage. Spearman’s rank correlation coefficients for each pair of phenotypes are indicated. The top associated marker for milk fat percentage in the mixed ancestry population is indicated on each plot as a blue dot (ARS-BFGL-NGS-57448; chr27 g.36155097C>T).

To examine the expression of genes at the chromosome 27 milk fat percentage locus, high-depth mammary RNAseq data from 212 lactating cows was assessed. Of the ten genes annotated in a 1 Mbp interval centred around the top milk fat percentage-associated SNP in the MA population (ARS-BFGL-NGS-57448; chr27 g.36155097C>T ±500 kbp), only *SFRP1*, *GOLGA7*, *AGPAT6*, and *KAT6A* were appreciably expressed (Figure 2C). Of these four genes, *AGPAT6* was the most highly expressed, with between 10 and 25-fold greater mean fragments per kilobase of exon model per million mapped (FPKM) expression values. Notably, the Cufflinks-predicted transcript structure of *AGPAT6* differed to the Entrez RefSeq annotation (NM_001083669), containing an additional exon approximately 14 kbp upstream of the NM_001083669 transcription start site (Figure 2D).

**Figure 3:**
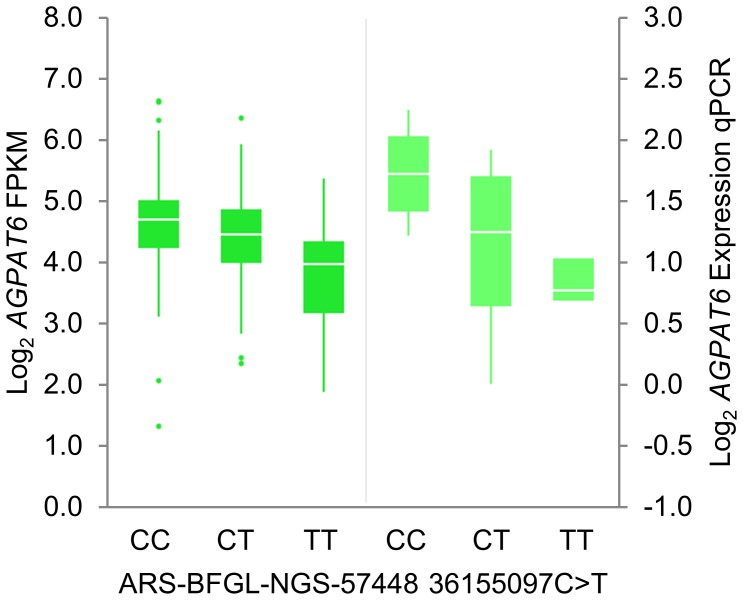
Box plots for *AGPAT6* expression by QTL genotype. Box plots showing expression of the *AGPAT6* gene in lactating mammary tissue quantified by RNA sequencing and quantitative PCR in conjunction with ARS-BFGL-NGS-57448 g.36155097C>T genotype. Log_2_ transformed fragments per kilobase of exon model per million mapped (FPKM) values derived by RNA sequencing are indicated on the left-hand Y-axis, log_2_ transformed relative expression values derived by quantitative PCR are on the right-hand axis. Genotype groups for the ARS-BFGL-NGS-57448 SNP are on the x-axis, p-values for association are P = 8.97×10^−7^ for RNA sequence data and P = 1.20×10^−3^ for quantitative PCR data.

Association analysis of the expression of these four genes was conducted using log_2_-transformed FPKM values in conjunction with the 281 Illumina BovineHD SNPs located in the 1 Mbp interval. This analysis revealed strong eQTL for both the *SFRP1* (P = 5.14×10^−9^) and *AGPAT6* (P = 4.69×10^−8^) genes (Figure 2A; Table S6). Association analysis was also conducted with milk fat percentage in the 1 Mbp interval in FJX animals (Figure 2A). Rank-correlation analysis of SNP p-values between the milk fat percentage QTL and eQTL association results was then conducted to assess genetic similarities of association between phenotypes. This analysis showed the p-value ranking for the *AGPAT6* eQTL and milk fat percentage QTL were highly correlated (R^2^ = 0.83; P = 2.38×10^−19^), whereas p-values for the milk fat percentage QTL and *SFRP1* eQTL were not correlated (R^2^ = 0.22; P = 0.06; Figure 2E). Quantitative PCR analysis of *AGPAT6* gene expression was also conducted using mammary RNA samples from 25 lactating cows, overlapping with the set used for RNAseq analysis. Association analysis using qPCR data in conjunction with ARS-BFGL-NGS-57448 genotype confirmed a significant eQTL for *AGPAT6* transcript abundance (P = 1.20×10^−3^). Figure 3 shows box and whisker plots of *AGPAT6* gene expression for ARS-BFGL-NGS-57448 genotype groups for both qPCR and RNAseq data. The allelic direction of effect was the same across both expression technologies, with the high-expression ARS-BFGL-NGS-57448 ‘C’ allele being the same allele associated with increased milk fat percentage in FJX and MA animals.

### Typing and Association Analysis of Novel *AGPAT6* Variants Demonstrates Strong Association with Milk Fat Percentage in FJX Animals

**Figure 4:**
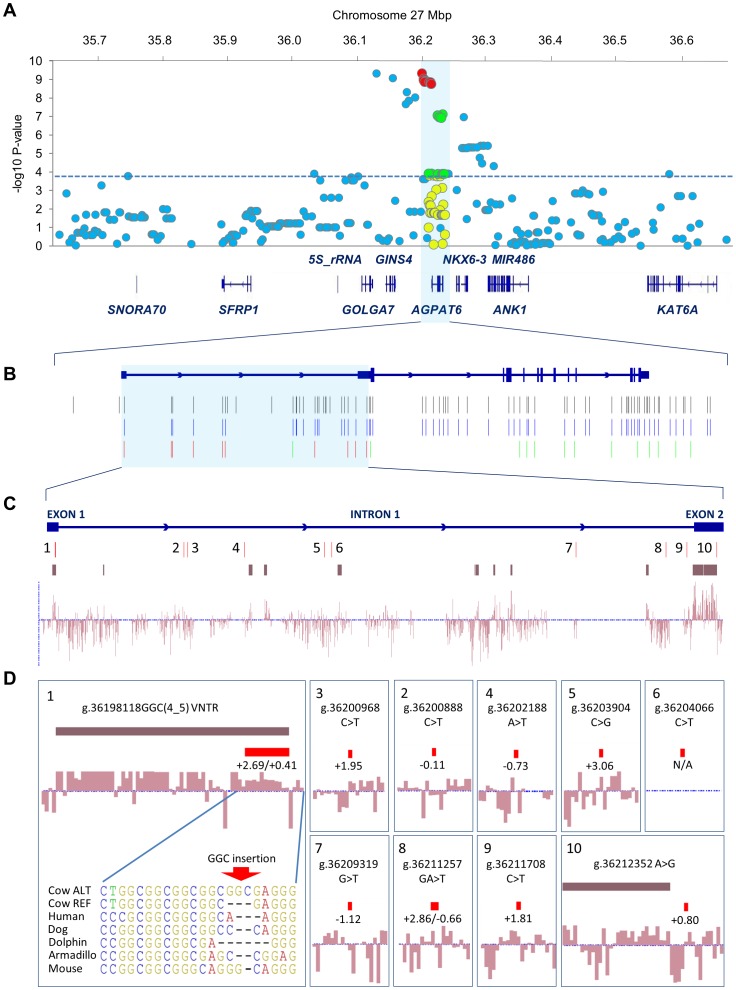
Analysis of *AGPAT6* candidate causative variants. Manhattan plot showing association of variants with milk fat percentage in Friesian-Jersey crossbreed animals in the chromosome 27 1 Mbp interval of interest (Figure 4A). The X-axis shows Mbp position on chromosome 27, the Y-axis shows –log_10_ p-values of marker association. Illumina BovineHD panel SNPs are indicated by blue dots. Custom-genotyped *AGPAT6* variants are indicated by red (highly associated), green (associated), and yellow (un-associated) dots, with significance of other markers demarcated by the dashed blue line. Figure 4B shows the distribution of F1 sire variants across the *AGPAT6* gene. The black variant track shows all variants targeted for genotyping from analysis of whole genome sequence data, the blue variant track shows genotyped variants that passed quality filtering criteria prior to association analysis. The red and green variant track shows significantly associated variants as displayed in Figure 4A. Figure 4C shows the distribution of highly associated variants with genome evolutionary rate profiling (GERP) conservation scores calculated from multiple genome alignments of 35 eutherian mammals. Site-wise scores are indicated by the light purple bar graph, with positive scores indicating conservation. Evolutionarily constrained elements are indicated by dark purple blocks, calculated from the same 35-way alignments. Figure 4D shows exploded views for each variant, with individual GERP scores indicated. For indel variants, the most and least conserved scores for the corresponding sequence are shown. An interspecies regional alignment for the g.36198118GGC(4_5) VNTR polymorphism is also shown.

The *AGPAT6* gene was examined further as the potential causative gene underlying the chromosome 27 milk fat percentage QTL. Analysis of whole genome sequence data from the six F1 sires at the heart of the FJX pedigree revealed 77 *AGPAT6* target variants for genotyping in the FJX F2 population. Genotyping and quality filtering of these genotypes yielded 59 variants, and these were added to the 1 Mbp interval of imputed Illumina BovineHD panel SNPs to yield 332 unique variants for association analysis. This analysis revealed strong association of novel *AGPAT6* variants with milk fat percentage in the FJX F2 animals (Figure 4A; Table S6). A biallelic 3 bp repeat expansion variant in *AGPAT6* 5′UTR exon 1 was the most significant variant in the interval (g.36198118GGC(4_5) VNTR; P = 4.81×10^−10^), though the ARS-BFGL-NGS-57448 and BovineHD2700010321 chip-SNPs, and nine sequence-derived *AGPAT6* variants had similar association statistics, and were in strong linkage disequilibrium (LD) with the g.36198118GGC(4_5) VNTR (R^2^>0.95). Using the *AGPAT6* gene structure determined from mammary RNA sequence data, all ten sequence-derived *AGPAT6* variants mapped to the 5′UTR regions of exons 1 and 2, and the connecting intron 1 sequence (Figure 4B & 4C). Site-wise genome evolutionary rate profiling (GERP) scores calculated from 35-way mammalian genome alignments indicated that these ten variants were evolutionarily conserved to varying degrees, though the g.36198118GGC(4_5) VNTR also coincided with a highly conserved element (Figure 4D). An additional 13 less highly associated *AGPAT6* variants were also statistically significant, with one of these mapping to the 3′UTR region of exon 13, and the others mapping to intronic or downstream sequence (Figure 4B; Table S7). The g.36198118GGC(4_5) VNTR explained 7.8% of the genetic variance and 5.9% of the phenotypic variance in milk fat percentage in the FJX F2 animals. Relative to g.36198118GGC(5) homozygous individuals, this translated to a 3.0% increase in milk fat percentage per high-fat GGC(4) allele. Individually fitting the g.36198118GGC(4_5) VNTR, the ARS-BFGL-NGS-57448 or BovineHD2700010321 SNPs, or any of the nine highly correlated *AGPAT6* variants as a fixed effect in the phenotypic model removed the significance of all other SNPs in the 1 Mbp interval.

## Discussion

We report a major QTL affecting milk fat percentage on bovine chromosome 27, in line with previous findings at this locus [10], [11]. Our analysis also suggests pleiotropic effects on a wide range of other milk components, including the individual proportions of milk fatty acids, milk volume, and protein and lactose content and yield. Using RNA sequencing and qPCR analysis of mammary tissue from lactating cows, we provide the first functional evidence of a causal role for *AGPAT6* underpinning these QTL, demonstrating differences in *AGPAT6* transcript abundance in conjunction with QTL genotype. We also report strong association of novel *AGPAT6* sequence variants with milk fat percentage, highlighting a subset of candidate causal polymorphisms in the 5′ UTR exons and intron 1 of the *AGPAT6* gene.

The milk fat percentage QTL on chromosome 27 was the second largest genome-wide QTL in both populations studied. Point-wise analysis using pedigree-based mixed models indicated that the ARS-BFGL-NGS-57448 SNP accounted for 1.6% and 7.8% of the genetic variance, and 1.3% and 5.7% of the phenotypic variance in MA and FJX animals respectively. The difference between these values across populations likely reflects the large difference in population size and allelic diversity represented by the MA and FJX animals, as well as differences in QTL allele frequency. Greater effects still were demonstrated for the well-studied DGAT1 locus on chromosome 14. Compared to analyses of most quantitative traits in humans where effect sizes are typically small, these observations further demonstrate that the genetic architecture of milk fat percentage in the cow models that of other bovine physiological traits such as stature [15], where single loci can account for large effects and large proportions of the total genetic and phenotypic variance. Quantitative trait loci affecting milk fat percentage, yield, and composition have been previously reported for the distal end of chromosome 27, detected using linkage analysis [15], and more recently GWAS [9]–[11]. It is interesting to note however that in recent genome scans of US Holstein-Friesian cattle [16], [17], large effects at this locus were not observed. New Zealand Holstein-Friesians comprised a major proportion of animals in our outbred population, and although US Holstein ancestry was represented in these animals, the QTL allele frequency in cattle of exclusive US ancestry is unknown. The large number of Jerseys will have assisted QTL detection in our population however, since the Holstein-Friesian and Jersey breeds in our sample carry opposing major alleles for this QTL. Given the commercial importance of the milk composition phenotypes associated with *AGPAT6* locus polymorphisms, it would be of interest to assess the frequency of tagging variants for this QTL in other cattle populations, since use of these variants may assist selection in dairy cattle breeding schemes.

The *AGPAT6* gene has been previously proposed as a candidate gene underlying milk fat composition QTL [9]–[11], though no functional data has been presented to support these claims. Two pieces of evidence from our analysis of mammary expression data support the status of *AGPAT6* as the causative gene underlying these QTL. First, of the four genes expressed in the 1 Mbp interval encompassing the milk fat percentage QTL, *AGPAT6* was by far the most abundantly expressed (∼10–25 fold greater mean FPKM). Second, and more definitively, we detected a strong *cis* eQTL for *AGPAT6* in this interval. Interestingly, a highly significant eQTL was also detected for the *SFRP1* gene. To assess whether either of these eQTL might underlie the milk fat percentage QTL, SNP p-value rank correlations were performed for gene expression and milk fat percentage association results. This analysis shows that the milk fat percentage and *AGPAT6* expression QTL are highly correlated, sharing the most significantly associated (and non-associated) SNPs in common. Although the *SFRP1* eQTL broadly coincides with the QTL for milk fat percentage, the genetic signature underlying *SFRP1* expression appears to be different than that for milk fat percentage, supporting the status of *AGPAT6* as the likely causative gene underlying this effect.

Irrespective of mammary expression evidence, *AGPAT6* also makes an excellent candidate gene on the basis of its own, relatively well-described biology. Most milk lipids are triglycerides, with triglyceride synthesis proceeding through the stepwise addition of fatty acyl groups to glycerol-3-phosphate. The AGPAT family of enzymes catalyse an intermediate step in this chain, converting 1-acylglycerol-3-phosphate to 1,2-diacylglycerol-3-phosphate [18]. Multiple AGPAT genes have so far been identified, but *AGPAT6* appears to be the most abundantly expressed isoform in the bovine mammary gland, with its expression correlating strongly with stage of lactation [13]. Notably, knockout of the *AGPAT6* gene in the mouse results in a reduction in the size and number of milk lipid droplets in mammary cells and ducts, with diacylglycerol and triacylglycerol content significantly reduced [14]. In the context of these findings, a straightforward hypothesis as to the mechanism underlying the milk fat percentage QTL proposes the following: that a genotype-driven increase in mammary *AGPAT6* gene expression results in a corresponding increase in *AGPAT6* enzyme abundance, resulting in increased milk triglyceride synthesis.

In addition to looking at effects on crude milk fat percentage for the *AGPAT6* locus, we also investigated possible effects on the underlying composition of milk fatty acids. The most significant finding from this analysis was an association with the relative proportion of C16 fatty acids, an effect that has been noted previously for this locus [9]. The same allele that was associated with increased *AGPAT6* gene expression and total milk fat percentage was associated with increased proportions of C16 fatty acids. This result could reasonably be expected, since the saturated fatty acid C16∶0 is the most predominant fatty acid in milk, about half of which is produced by *de novo* triglyceride synthesis in the mammary gland [19]. We also observed a significant increase in saturated fatty acid content, a result that can be partially explained by the increase in C16∶0. The high milk fat percentage allele was associated with decreases in most of the other fatty acids, suggesting competition in synthesis between C16 and the other mammary-gland derived fatty acids, and potentially some form of homeostatic mechanism for the long-chain dietary-derived lipids. We also found significant effects for the *AGPAT6* locus on lactose percentage and yield, protein percentage and yield, and milk volume. Although not all associations were significant in both the MA and FJX populations, in all cases, the allelic direction of effect was the same across populations, with increased milk fat percentage accompanying increased milk fat yield and protein percentage, and decreased lactose yield and percentage, milk volume, and milk protein yield. Since differences in *AGPAT6* expression presumably only directly impact fat synthesis, these pleiotropic effects must be coupled through secondary mechanisms of milk composition regulation. Lactose is a key osmo-regulator in milk [20], so a reduction in lactose synthesis would be expected to lead to a reduction in milk volume, and a corresponding increase in milk protein percentage. The interplay between these mechanisms is unknown, though remarkably, the traits affected and the allelic direction of effects are identical to those attributed to variants of the bovine *DGAT1* gene [6]. Both *AGPAT6* and *DGAT1* are key acyltransferase enzymes of the mammary triglyceride synthesis pathway, being separated by a single node in this chain [12]. The analogous roles of *AGPAT6* and *DGAT1* might suggest that these pleiotropic effects on milk composition are a response peculiar to alterations in the rate of triglyceride synthesis, however we have observed similar pleiotropic effects for other major milk components. As an example, the fourth largest milk fat percentage effect estimated in our GWAS of 32,530 cows was for the 103–104 Mbp interval on chromosome 11 (Table S2), representing a region that encompasses a well-documented QTL for the major milk protein beta-lactoglobulin [21]. Taken together, these observations suggest that there are broad, interrelated regulatory mechanisms governing the yield and concentration of major milk components, and that a change in one of these components leads to a reconfiguration of total milk composition. Interestingly, the effects of *AGPAT6* variants may also impact lipid synthesis outside of the mammary gland, with the same chromosome 27 QTL region also implicated in back fat thickness and marbling in beef cattle [22]–[24], effects that have been observed for *DGAT1* polymorphisms [25], [26]. This proposition is further supported by the observation that, in addition to defects in mammary lipid synthesis, *AGPAT6* knockout mice also have significantly reduced adipose tissue mass [27].

By analysing whole genome sequence data representing the six F1 sires of the FJX pedigree, we aimed to identify candidate causative variants underlying the milk fat percentage and *AGPAT6* expression QTL. Non-synonymous coding variants were not identified in these animals, consistent with these QTL being underpinned by an expression-based mechanism. Genotyping and association analysis of 59 *AGPAT6* locus variants in the FJX F2 population revealed strongest associations for a subset of 10 variants spanning *AGPAT6* 5′UTR exons 1 and 2, and the connecting intron. Fitting any one of these variants as an effect in the milk fat percentage phenotypic model removed significance for all other variants at this locus, suggesting that these variants fully account for the milk fat percentage QTL at this locus. The *AGPAT6* gene was recently proposed as a candidate gene for milk fat percentage in a GWAS of German Holstein-Friesian animals [10]. The authors of that study targeted two *AGPAT6* variants for genotyping in their study population, and demonstrated association for a substitution variant g.36211257GA>T. On the basis of transcription factor binding site prediction, the authors speculated a functional role for the g.36211257GA>T variant. Interestingly, the g.36211257GA>T substitution is one of the ten highly associated variants identified in the current study, though based on the transcript structure identified by mammary RNAseq analysis, this variant falls within intron 1 of *AGPAT6,* as opposed to upstream of the transcription start site. This does not preclude a *cis*-regulatory role for the g.36211257GA>T variant, however on the basis of our analysis, the GGC repeat expansion g.36198118GGC(4_5) in exon 1 seems the most likely causal candidate. This insertion maps to an evolutionarily constrained element identified by conservation analysis of 35-way mammalian alignments, and coincides with a number of ENCODE transcription factor binding sites and open chromatin signals [28] for the syntenic region of human *AGPAT6* exon 1. These observation aside, without functional testing it cannot be determined whether the g.36198118GGC(4_5) insertion, g.36211257GA>T substitution, or any of the other associated variants have any biological impact on *AGPAT6* gene expression and milk fat synthesis in the mammary gland. We assessed a 5 kbp region of sequence upstream of the *AGPAT6* transcription start site for candidate causal variants for FJX genotyping. Attempts to genotype two upstream variants that were in strong LD with the ARS-BFGL-NGS-57448 SNP, and therefore likely to be statistically associated with *AGPAT6* expression and milk fat percentage, failed to yield genotypes of sufficient quality for analysis. Although neither of these variants are evolutionarily conserved, it is also possible that one of these, or other untested variants at the broader *AGPAT6* locus may be causal for these QTL.

In summary, we report investigation of a major QTL affecting diverse milk composition phenotypes on bovine chromosome 27. We provide transcriptomic evidence suggesting that expression variants of the *AGPAT6* gene likely underlie these effects, and propose a number of candidate causative polymorphisms for future analysis and testing. It is noteworthy from our analysis that the two largest genome-wide effects on milk fat percentage implicate two genes that are separated by a single node in the triglyceride synthesis pathway. Future work will examine the genes and genetic relationships of other members of this pathway, and help shed further light on the genetic underpinnings of milk composition regulation.

## Methods

### Ethics Statement

Animal ethics approval was granted for all animal work by the Ruakura Animal Ethics Committee, Hamilton, New Zealand. No animals were sacrificed for this study. Mammary tissue samples were obtained by needle biopsy in accordance with protocols approved by the ethics committee (approval AEC 12845).

### GWAS Animal Populations

The cross-breed pedigree consisted of a subset of 864 F2 New Zealand Holstein-Friesian × Jersey (FJX) dairy cows representing the half-sib daughters of six F1 sires [29]. This pedigree was comprised of two cohorts bred over successive years and located on the same research farm, which is a resource that has been used previously for the discovery of QTL and causative genes and variations [30]–[32]. The mixed ancestry (MA) population consisted of 32,530 female dairy cows located on commercial dairy farms throughout New Zealand, forming part of a large phenotypic and genotypic database of animals used for evaluation of sire performance. These animals were born between 1994 and 2010, with ∼99% of records (32,120) derived from animals born after 2004. The MA cows consisted of 11,096 NZ Holstein-Friesians, 8,096 Jerseys, and 13,338 NZ Holstein-Friesian × Jersey cross breeds in the case where pure Holstein-Friesians and Jerseys were defined as having a breed proportion of at least 13/16ths. Minor proportions of other breeds were also present in these animals, including Ayreshire, Brown Swiss, Guernsey, Hereford, Milking Shorthorn, Swedish Red, and others.

### Milk Composition Analysis

The concentrations of major milk components were measured as part of standard herd-testing procedures using Fourier transform infrared spectroscopy. Most milk samples were processed by LIC Testlink (Newstead, Hamilton, New Zealand) using the MilkoScan FT6000 instrument (FOSS, Hillerød, Denmark). A subset of samples representing the FJX animals were alternatively processed by DairyNZ (Newstead, Hamilton, New Zealand) using the MilkoScan FT120 instrument (FOSS). For animals in the MA population (32,530 animals), milk composition records were restricted to first lactation measurements from October to January inclusive. For FJX F2 cows (711 animals), herd test results from the animals’ second lactation were used, representing a three month period in mid-lactation (November to January in the 2004 and 2005 seasons for cohort 1 and 2 respectively). Second lactation data was also used for fatty-acid analysis of milk fat in FJX animals, with milk samples taken at peak (September/October), mid (November) and late lactation (February). Fatty acids were extracted by a modification of the Röse Gottlieb technique [33], and quantified by gas-liquid chromatography on a Shimadzu GC17A instrument (Shimadzu Corporation) at Fonterra Research Centre in Palmerston North, New Zealand. The relative proportions of individual fatty acids were calculated as grams per 100 g of total fatty acid, but for simplification of presentation, groups of individual fatty acids were also combined into categories. See Methods S1 for detailed descriptions of animal phenotype inclusion criteria, herd testing details, and fatty acid categorisation and chromatography methods..

### High Throughput Genotyping and Variant Imputation

Genomic DNA extraction for the FJX, MA, RNAseq, and qPCR animals is described in Methods S1. All high throughput genotyping was conducted by GeneSeek (Lincoln, NE, USA). FJX and MA animals were typed using the Illumina BovineSNP50 BeadChip (Illumina), with a proportion of MA animals also typed using the Illumina BovineHD BeadChip. Both FJX and MA populations were then imputed to 711,678 BovineHD BeadChip SNPs using Beagle software (Beagle v3.3.2) [34], using a reference population of 3,206 animals. Animals that were biopsied for RNAseq in 2013 were also genotyped using the Illumina BovineHD BeadChip. For the 12 non-FJX animals that were analysed by qPCR, the Illumina BovineHD BeadChip SNP ARS-BFGL-NGS-57448 was manually genotyped using PCR and Sanger sequencing, as described in Methods S1.

### RNA Sequencing

High-depth RNAseq was undertaken using mammary gland tissue samples from 217 lactating cows. These samples comprised three subgroups sampled at different points through time. One hundred and eighty-eight biopsies were taken in January 2013, sampled from Holstein-Friesian cows in their second or third lactation and producing 10–17 L of milk per day. Twenty-nine of the samples came from F2 animals from the same FJX pedigree used for genetic association analysis, with 12 of these producing 7–15 L of milk per day and sampled in March 2012, and 17 of these producing 6–15 L of milk per day sampled in April 2004 as part of a separate study [35]. Tissue samples were taken by needle biopsy for all animals as previously described [35], and total RNA was extracted by NZ Genomics Limited (NZGL; Auckland, New Zealand) as detailed in Methods S1. For the 29 RNA samples derived from FJX F2 animals, libraries were prepared using the TruSeq RNA Sample Prep Kit v2 (Illumina), and sequenced by NZGL (Dunedin, New Zealand) using the Illumina HiSeq 2000 instrument. For the 188 samples taken in 2013, libraries were prepared using the TruSeq Stranded Total RNA Sample Prep Kit (Illumina) with ribosomal RNA depletion using the Human/Mouse/Rat Ribo-Zero kit (Epicentre/Illumina). These samples were sequenced by the Australian Genome Research Facility (AGRF; Melbourne, Australia) using the Illumina HiSeq 2000 instrument. Sequence data were mapped to the UMD 3.1 genome using Tophat2 (version 2.0.8) [36], locating an average of 87 million read-pairs for the 29 FJX animals, and 84 million pairs for the 188 Holstein-Friesian animals. Cufflinks software (version 2.1.1) [37] was used to quantify expressed transcripts, and yielded fragments per kilobase of exon model per million mapped (FPKM) expression values. For the 10 genes in the chromosome 27 1 Mbp region of interest, genes were considered for downstream analysis if they had non-zero FPKM values in at least 75% of samples, and had a mean expression of 0.5 FPKM or greater. Methods S1 outlines read mapping and transcript calling parameters in greater detail.

### *AGPAT6* Quantitative RT-PCR

Biopsies from lactating mammary tissue were taken from animals as previously described [35]. A total of 25 samples from 25 animals were used for qPCR analysis, with 13 of these taken from FJX F2 animals, eight of which overlapped with the set used for RNA sequencing (described above). The remaining 12 samples were taken from unrelated mixed-age Holstein Friesian and Holstein Friesian-Jersey crossbred cows at mid-lactation in January 2004 (approximately 150 days post-partum). Total RNA was extracted and integrity assessed as previously described [35]. Complementary DNA was synthesised using the Superscript III Supermix kit (Invitrogen). A custom intron-spanning assay targeting exons 7 & 8 of the *AGPAT6* transcript reference sequence (NM_001083669.1) was designed using Roche Universal Probe Library software (Roche). Two additional assays targeting EIF3K and RPS15A genes were designed to serve as endogenous controls for PCR normalisation. Quantitative PCR was conducted using the Roche Lightcycler 480 instrument (Roche Diagnostics). Relative quantification was performed by dividing mean *AGPAT6* concentration values by mean endogenous control gene values, yielding normalised ratios of *AGPAT6* transcript to each endogenous control gene for each sample. The geometric mean of these values was then normalised to a calibrator sample to transform data to positive values for downstream data presentation and statistical analysis. Further information regarding cDNA synthesis, qPCR assay design, amplification conditions and other qPCR experimental parameters is detailed in Methods S1.

### Genome Sequence Analysis and Custom Genotyping

High-depth whole genome sequencing was conducted on the six F1 males of the FJX pedigree as described previously [32]. Sequence read mapping and variant-calling for these data is described in Methods S1. All F1 sire variants within an approximately 40 kb interval encompassing the *AGPAT6* gene were extracted for further analysis. This interval contained 20 kb of 5′ upstream sequence and 5 kb of 3′downstream sequence relative to the *AGPAT6* RefSeq entry NM_001083669.1 gene co-ordinates. Twenty kilobases of upstream sequence was targeted since this encompassed an alternative *AGPAT6* first exon that was not included in the Entrez Gene annotation (NM_001083669.1), though evidenced by EST data (e.g. GenBank EST AV610098), and RNAseq data from lactating mammary samples. This 40 kb genomic interval therefore included 5 kb upstream and downstream of the mammary-expressed transcript, and contained 121 low quality-threshold variants generated from automated calling of sequence data. A subset of 76 of these variants (73 SNPs and three indels) was targeted for Sequenom iPLEX assay design and genotyping by GeneSeek (Lincoln, NE, USA). Methods S1 and Table S7 detail the filtering steps from raw variants to manually curated target variants. Sequence.BAM files representing the target region in the six F1 sires can be accessed at the NCBI Sequence Read Archive (http://www.ncbi.nlm.nih.gov/sra), accession number SRP033212. The high priority indel variant chr27 g.36198118GGC(4_5) VNTR could not be typed using the Sequenom platform so was instead targeted using a fluorescent PCR-based assay. Details for this assay are in Methods S1.

### Statistical Analysis

#### Primary data

Genotype, phenotype, and pedigree data used for analysis in the MA, FJX, RNAseq, and qPCR populations have been deposited in the Dryad digital data repository (http://datadryad.org/; doi:10.5061/dryad.d6508).

#### Bayesian genome-wide association analysis in the MA and FJX populations

A minor allele frequency (MAF) filter of 0.1% was applied to the union of MA and FJX genotype data yielding 653,725 SNPs across the 32,530 MA and 711 FJX animals. Genome-wide association analysis was conducted using GenSel software (Version 4.53R) [38]. Markers were fit simultaneously by running a Bayes B model [39] for 50,000 iterations, including a burn-in of 20,000 iterations. R-inverse weights were applied according to the reliability of the phenotype, and covariates for birth year, proportion NZ Holstein-Friesian ancestry, proportion US Holstein-Friesian ancestry, and proportion Jersey ancestry were also included in these models. Bayes B genetic and residual variance priors were estimated by running Bayes Cpi with pi = 0.95 for 20,000 iterations, including a burn-in of 10,000 iterations. In addition to considering effects for individual markers, the genome was also partitioned into 1 Mbp windows and the combined effects of SNPs within these intervals were estimated.

#### Genetic association analysis of *AGPAT6* locus variants

Sequence-derived *AGPAT6* variants were analysed together with imputed Illumina BovineHD SNPs in a 1 Mbp interval on chromosome 27. This interval centred around the top associated SNP identified by Bayes B analysis in the MA population (ARS-BFGL-NGS-57448; chr27 g.36155097C>T ±500 kbp). Prior to association analysis, custom genotyping data were filtered according to a number of quality criteria, yielding 59 variants for analysis. Methods S1 details the criteria used for filtering, Table S7 lists these variants. For Illumina BovineHD SNPs, 281 variants (pre-filtered using the same criteria used for genome-wide association analysis) mapped to the interval, with eight of these variants excluded due to redundancy with custom-genotyped SNPs (yielding 273 SNPs; Table S6). Associations between SNPs and the milk fat percentage phenotype were quantified by restricted maximum likelihood (REML) using pedigree-based mixed models in ASReml-R [40], [41]. Each SNP was fitted in a separate model, with SNP treated as a quantitative variable based on the number of allele copies. Pedigree relationships were accounted for by fitting a pedigree-based relationship matrix, and a fixed effect for birth year was also included in each model. Variants were considered significant at P<1.51×10^−4^, incorporating a Bonferroni correction for multiple hypothesis testing using an alpha = 0.05. For point-wise analysis of the ARS-BFGL-NGS-57448 SNP in conjunction with other gross milk composition phenotypes in the FJX and MA populations, the same model components were used as described above. For repeated measures analysis of milk fatty acids in FJX animals, models additionally included stage of lactation as a fixed effect (peak, mid, late), animal as a random effect, and days from the mean herd calving date as a covariate. The proportion of phenotypic variance explained by each SNP for each phenotype was calculated using (2p(1-p)a^2^)/t, where *p* is the frequency of the A allele, *a* is the estimated allele substitution effect, and *t* is the total phenotypic variation. Similarly, the proportion of genetic variance explained by each SNP was calculated using (2p(1-p)a^2^)/g, where *g* is the total genetic variance.

#### *AGPAT6* eQTL analysis – RNA sequencing

For the 217 mammary biopsy samples that were sequenced, Illumina BovineHD BeadChip genotypes were available on 212 individuals. Four of the ten genes in the chromosome 27 1 Mbp interval of interest passed the nominal expression threshold for analysis (see RNA Sequencing section above). Association analysis was conducted using log_2_-transformed FPKM values and the same 281 Illumina BovineHD BeadChip SNPs used for analysis of FJX milk fat percentage, quantified by restricted maximum likelihood (REML) using pedigree based mixed models in ASReml-R [40], [41]. Each SNP was treated as a quantitative variable, biopsy year and sequencing facility were included as fixed effects, and pedigree relationships were accounted for by fitting a pedigree-based relationship matrix. Variants were considered significant at P<1.80×10^−4^, incorporating a Bonferroni correction for multiple hypothesis testing using an alpha = 0.05.

#### *AGPAT6* eQTL analysis - quantitative RT-PCR

Association analysis between *AGPAT6* qPCR transcript abundance and *AGPAT6* locus genotype was conducted by fitting an lm model in R (v3.0.1) [42]. Relative *AGPAT6* gene expression was transformed using a log_2_ transformation and ARS-BFGL-NGS-57448 was treated as a quantitative variable based on the number of allele copies. Biopsy group was fitted as a fixed effect to account for potential differences in expression between the two different biopsy time-points.

#### QTL correlation analysis

To provide insight into whether the co-locating QTL for milk fat percentage and gene expression might have common genetic underpinnings, correlation analysis was conducted using the lists of SNP association statistics between traits. This approach postulates that two QTL underpinned by a single causative variant will produce similar patterns of association for a given set of SNPs (or rather the haplotypes that they tag), whereas two QTL underpinned by discrete variants on different haplotypes will generate a different association signature, and different associated SNP ranking. By this logic, the relative ranking of these SNPs should correlate between co-regulated traits, and be uncorrelated between independently regulated traits. In reality, the haplotype structure underlying any two co-located QTL is unlikely to give rise to truly independent signals, and QTL may be correlated due to shared haplotypes alone. For comparative purposes, however, as in the case of comparing *cis* eQTL of candidate causative genes with physiological QTL, the degree of correlation may allow differentiation between candidate genes. In the current application, we selected all SNP from a 250 kbp window centred around the top milk fat percentage associated SNP identified by the Bayes B analysis for the MA population (ARS-BFGL-NGS-57448; chr27 g.36155097C>T ±125 kbp). This window size was selected to proportionally increase the number of SNPs positively associated with milk fat percentage in the correlation set, since although non-associated SNPs also provide differentiation in correlation between QTL, an excess of non-associated SNPs would decrease the sensitivity of rank correlation. Spearman’s rank correlation coefficients were then calculated for the milk fat percentage and eQTL p-values for the 72 SNPs in this interval. Nonparametric correlation analysis was performed since the relative sizes of the p-values are not necessarily numerically comparable between different QTL. It should also be noted that different populations of animals were used in this analysis, so this method also assumes shared haplotypes across populations.

### Conservation Analysis of Candidate Causative Variants

The degree of evolutionary conservation was assessed for sequence-derived *AGPAT6* variants that were highly associated with milk fat percentage. Site-wise genomic evolutionary rate profiling (GERP) [43] scores were retrieved for these variants from whole-genome multiple alignments of 35 eutherian mammals, calculated against the Btau4.0 bovine genome and visualised using the Ensembl genome browser [44]. Constrained elements were also retrieved for these regions, defined as stretches of the multiple alignment where sequences are highly conserved based on the previous GERP score. The Ensembl browser was also used to visualise ENCODE [28] open chromatin and transcription factor binding sites for human *AGPAT6*. Multiple species alignments for *AGPAT6* exon 1 were conducted using Clustal W [45], using cow, human, dog, dolphin, armadillo, and mouse genomic sequences.

## Supporting Information

Table S1 Individual SNP effects from genome-wide association analysis in the MA population. ‘Mkr_ID’ indicates the Illumina BovineHD SNP name, ‘Effect’ indicates the allele substitution effect estimated by Bayes B. This file is a modified output from the GenSel analysis package, see Fernando & Garrick [38] for a detailed description of other output fields.
(XLSX)

Table S2 Top 50 milk fat percentage QTL identified by window analysis in MA animals, where the variances of consecutive 1 Mbp windows of SNPs throughout the genome are estimated by Bayes B. This file is a modified output from the GenSel analysis package, see Fernando & Garrick [38] for a detailed description of output fields.
(XLSX)

Table S3 Individual SNP effects from genome-wide association analysis in the FJX population. ‘Mkr_ID’ indicates the Illumina BovineHD SNP name, ‘Effect’ indicates the allele substitution effect estimated by Bayes B. This file is a modified output from the GenSel analysis package, see Fernando & Garrick [38] for a detailed description of other output fields.
(XLSX)

Table S4 Top 50 milk fat percentage QTL identified by window analysis in FJX animals, where the variances of consecutive 1 Mbp windows of SNPs throughout the genome are estimated by Bayes B. This file is a modified output from the GenSel analysis package, see Fernando & Garrick [38] for a detailed description of output fields.
(XLSX)

Table S5 Individual fatty acid association statistics for the ARS-BFGL-NGS-57448 SNP. ‘PhenName’ indicates the individual fatty acids tested, ‘n’ is the number of animals used for analysis. ‘Parameter_estimate’ and ‘Parameter_se’ indicate the per-allele parameter estimate and standard errors calculated from the restricted maximum likelihood models, with parameter adjusted genotype group means also indicated. ‘Pheno_variance’ indicates the proportion of phenotypic variance explained by the ARS-BFGL-NGS-57448 SNP for each fatty acid tested, with p-values of association indicated in the right-most column.
(XLSX)

Table S6 Association statistics for variants within the 1 Mbp milk fat percentage QTL region. ‘Var_name_custom_and_HD’ indicates the merged custom-genotyped and Illumina BovineHD SNP-chip variant names, with ‘Var_name_HD’ indicating names of variants from the SNP-chip only. ‘Chr’, ‘bp’, and ‘Mbp’ indicate the positions of variants on the UMD3.1 genome build. For each block of association statistics for the milk fat percentage and *AGPAT6* and *SFRP1* expression phenotypes, the parameter estimates, the parameter estimate standard errors, p-values of association, and the percentage of phenotypic variance explained by each SNP are indicated. ‘Vars_for_corrs’ indicates the 250 kbp window of variants used for calculation of Spearman’s rank correlation coefficients for lists of p-values between phenotypes.
(XLSX)

Table S7 List of *AGPAT6* locus variants discovered by whole genome sequencing of the six FJX F1 sires, and subsequent curation and quality filtering steps for target variants. ‘#CHROM’, ‘POS’, ‘REF’, and ‘ALT’ indicate the position and reference and alternate alleles for each variant called by the automated variant callers, ‘QUAL’ indicates the quality score of each variant. ‘Assay_Curation’ indicates details of target variant selection, and the likely validity of variant calls following manual inspection of sequence alignments. ‘SNPEff_Effect’ shows the *in silico* functions predicted by SNPEff software, ‘Target_vars_corrected’ indicates the subset of variants targeted for genotyping in the FJX F2 animals. ‘Quality_filtered_vars_for_assoc’ lists the variants that were successfully genotyped and passed genotype quality threshold criteria, highlighted in yellow across all fields.
(XLSX)

Methods S1 **Supplementary methods.**
(DOCX)

## References

[1] Neville MC, Picciano MF (1997) Regulation of milk lipid secretion and composition. Annual review of nutrition 17: 159–183. 10.1146/annurev.nutr.17.1.159 9240924

[2] Heyen DW, Weller JI, Ron M, Band M, Beever JE, et al (1999) A genome scan for QTL influencing milk production and health traits in dairy cattle. Physiological genomics 1: 165–175. 11015574 10.1152/physiolgenomics.1999.1.3.165

[3] Pryce JE, Bolormaa S, Chamberlain AJ, Bowman PJ, Savin K, et al (2010) A validated genome-wide association study in 2 dairy cattle breeds for milk production and fertility traits using variable length haplotypes. Journal of Dairy Science 93: 3331–3345. 20630249 10.3168/jds.2009-2893

[4] Schopen GCB, Visker MHPW, Koks PD, Mullaart E, van Arendonk JAM, et al (2011) Whole-genome association study for milk protein composition in dairy cattle. Journal of Dairy Science 94: 3148–3158. 21605784 10.3168/jds.2010-4030

[5] Jiang L, Liu J, Sun D, Ma P, Ding X, et al (2010) Genome wide association studies for milk production traits in Chinese Holstein population. PloS one 5: e13661. 21048968 10.1371/journal.pone.0013661 PMC2965099

[6] Grisart B, Coppieters W, Farnir F, Karim L, Ford C, et al (2002) Positional candidate cloning of a QTL in dairy cattle: identification of a missense mutation in the bovine DGAT1 gene with major effect on milk yield and composition. Genome research 12: 222–231. 11827942 10.1101/gr.224202

[7] Schennink A, Stoop WM, Visker MHPW, Heck JML, Bovenhuis H, et al (2007) DGAT1 underlies large genetic variation in milk-fat composition of dairy cows. Animal genetics 38: 467–473. 17894561 10.1111/j.1365-2052.2007.01635.x

[8] Cases S, Smith SJ, Zheng YW, Myers HM, Lear SR, et al (1998) Identification of a gene encoding an acyl CoA:diacylglycerol acyltransferase, a key enzyme in triacylglycerol synthesis. Proceedings of the National Academy of Sciences of the United States of America 95: 13018–13023. 9789033 10.1073/pnas.95.22.13018 PMC23692

[9] Bouwman AC, Bovenhuis H, Visker MHPW, van Arendonk JAM (2011) Genome-wide association of milk fatty acids in Dutch dairy cattle. BMC genetics 12: 43. 21569316 10.1186/1471-2156-12-43 PMC3120725

[10] Wang X, Wurmser C, Pausch H, Jung S, Reinhardt F, et al (2012) Identification and Dissection of Four Major QTL Affecting Milk Fat Content in the German Holstein-Friesian Population. PloS one 7: e40711. 22792397 10.1371/journal.pone.0040711 PMC3394711

[11] Strucken EM, Bortfeldt RH, Tetens J, Thaller G, Brockmann GA (2012) Genetic effects and correlations between production and fertility traits and their dependency on the lactation-stage in Holstein Friesians. BMC genetics 13: 108. 23244492 10.1186/1471-2156-13-108 PMC3561121

[12] Coleman R (2004) Enzymes of triacylglycerol synthesis and their regulation. Progress in Lipid Research 43: 134–176. 14654091 10.1016/s0163-7827(03)00051-1

[13] Bionaz M, Loor JJ (2008) ACSL1, AGPAT6, FABP3, LPIN1, and SLC27A6 Are the Most Abundant Isoforms in Bovine Mammary Tissue and Their Expression Is Affected by Stage of Lactation. J Nutr 138: 1019–1024. 18492828 10.1093/jn/138.6.1019

[14] Beigneux AP, Vergnes L, Qiao X, Quatela S, Davis R, et al (2006) Agpat6–a novel lipid biosynthetic gene required for triacylglycerol production in mammary epithelium. Journal of lipid research 47: 734–744. 16449762 10.1194/jlr.M500556-JLR200 PMC3196597

[15] Zhang Q, Boichard D, Hoeschele I, Ernst C, Eggen A, et al (1998) Mapping quantitative trait loci for milk production and health of dairy cattle in a large outbred pedigree. Genetics 149: 1959–1973. 9691050 10.1093/genetics/149.4.1959 PMC1460288

[16] Cole JB, Wiggans GR, Ma L, Sonstegard TS, Lawlor TJ, et al (2011) Genome-wide association analysis of thirty one production, health, reproduction and body conformation traits in contemporary U.S. Holstein cows. BMC genomics 12: 408. 21831322 10.1186/1471-2164-12-408 PMC3176260

[17] Cole JB, VanRaden PM, O’Connell JR, Van Tassell CP, Sonstegard TS, et al (2009) Distribution and location of genetic effects for dairy traits. Journal of Dairy Science 92: 2931–2946. 19448026 10.3168/jds.2008-1762

[18] Agarwal AK (2012) Lysophospholipid acyltransferases: 1-acylglycerol-3-phosphate O-acyltransferases. From discovery to disease. Current opinion in lipidology 23: 290–302. 22777291 10.1097/MOL.0b013e328354fcf4

[19] Månsson HL (2008) Fatty acids in bovine milk fat. Food & nutrition research 52. 10.3402/fnr.v52i0.1821 PMC2596709 19109654

[20] Rook J (1979) The role of carbohydrate metabolism in the regulation of milk production. Proceedings of the Nutrition Society 38: 309–314. 575218 10.1079/pns19790053

[21] Berry SD, Lopez-Villalobos N, Beattie EM, Davis SR, Adams LF, et al (2010) Mapping a quantitative trait locus for the concentration of beta-lactoglobulin in milk, and the effect of beta-lactoglobulin genetic variants on the composition of milk from Holstein-Friesian x Jersey crossbred cows. New Zealand veterinary journal 58: 1–5. 20200568 10.1080/00480169.2010.65053

[22] McClure MC, Morsci NS, Schnabel RD, Kim JW, Yao P, et al (2010) A genome scan for quantitative trait loci influencing carcass, post-natal growth and reproductive traits in commercial Angus cattle. Animal genetics 41: 597–607. 20477797 10.1111/j.1365-2052.2010.02063.x

[23] Casas E, Shackelford SD, Keele JW, Stone RT, Kappes SM, et al (2000) Quantitative trait loci affecting growth and carcass composition of cattle segregating alternate forms of myostatin. Journal of animal science 78: 560–569. 10764062 10.2527/2000.783560x

[24] Nalaila SM, Stothard P, Moore SS, Li C, Wang Z (2012) Whole-genome QTL scan for ultrasound and carcass merit traits in beef cattle using Bayesian shrinkage method. Journal of animal breeding and genetics = Zeitschrift für Tierzüchtung und Züchtungsbiologie 129: 107–119. 22394233 10.1111/j.1439-0388.2011.00954.x

[25] Thaller G, Kuhn C, Winter A, Ewald G, Bellmann O, et al (2003) DGAT1, a new positional and functional candidate gene for intramuscular fat deposition in cattle. Animal Genetics 34: 354–357. 14510671 10.1046/j.1365-2052.2003.01011.x

[26] Li X, Ekerljung M, Lundström K, Lundén A (2013) Association of polymorphisms at DGAT1, leptin, SCD1, CAPN1 and CAST genes with color, marbling and water holding capacity in meat from beef cattle populations in Sweden. Meat science 94: 153–158. 23501244 10.1016/j.meatsci.2013.01.010

[27] Vergnes L, Beigneux AP, Davis R, Watkins SM, Young SG, et al (2006) Agpat6 deficiency causes subdermal lipodystrophy and resistance to obesity. Journal of lipid research 47: 745–754. 16436371 10.1194/jlr.M500553-JLR200 PMC2901549

[28] Bernstein BE, Birney E, Dunham I, Green ED, Gunter C, et al (2012) An integrated encyclopedia of DNA elements in the human genome. Nature 489: 57–74. 22955616 10.1038/nature11247 PMC3439153

[29] Spelman RJ, Miller FM, Hooper JD, Thielen M, Garrick DJ (2001) Experimental design for QTL trial involving New Zealand Friesian and Jersey breeds. Proceedings of the Association for the Advancement of Animal Breeding and Genetics 14: 393–396.

[30] Berry SD, Davis SR, Beattie EM, Thomas NL, Burrett AK, et al (2009) Mutation in bovine beta-carotene oxygenase 2 affects milk color. Genetics 182: 923–926. 19398771 10.1534/genetics.109.101741 PMC2710170

[31] Karim L, Takeda H, Lin L, Druet T, Arias JAC, et al (2011) Variants modulating the expression of a chromosome domain encompassing PLAG1 influence bovine stature. Nature genetics 43: 405–413. 21516082 10.1038/ng.814

[32] Berry S, Coppieters W, Davis S, Burrett A, Thomas N, et al (2013) A Triad of Highly Divergent Polymeric Immunoglobulin Receptor (PIGR) Haplotypes with Major Effect on IgA Concentration in Bovine Milk. PloS one 8: e57219. 23536764 10.1371/journal.pone.0057219 PMC3594236

[33] International Dairy Federation (1987) Milk. Determination of fat content – Rose Gottlieb gravimetric method.

[34] Browning BL, Browning SR (2009) A unified approach to genotype imputation and haplotype-phase inference for large data sets of trios and unrelated individuals. American journal of human genetics 84: 210–223. 19200528 10.1016/j.ajhg.2009.01.005 PMC2668004

[35] Littlejohn MD, Walker CG, Ward HE, Lehnert KB, Snell RG, et al (2010) Effects of reduced frequency of milk removal on gene expression in the bovine mammary gland. Physiological genomics 41: 21–32. 19996161 10.1152/physiolgenomics.00108.2009

[36] Kim D, Pertea G, Trapnell C, Pimentel H, Kelley R, et al (2013) TopHat2: accurate alignment of transcriptomes in the presence of insertions, deletions and gene fusions. Genome biology 14: R36. 23618408 10.1186/gb-2013-14-4-r36 PMC4053844

[37] Trapnell C, Williams BA, Pertea G, Mortazavi A, Kwan G, et al (2010) Transcript assembly and quantification by RNA-Seq reveals unannotated transcripts and isoform switching during cell differentiation. Nature biotechnology 28: 511–515. 10.1038/nbt.1621 PMC3146043 20436464

[38] Fernando RL, Garrick DJ (2010) GenSel - User manual for a portfolio of genomic selection related analyses: Animal Breeding and Genetics, Iowa State Universit.

[39] Meuwissen THE, Hayes BJ, Goddard ME (2001) Prediction of Total Genetic Value Using Genome-Wide Dense Marker Maps. Genetics 157: 1819–1829. 11290733 10.1093/genetics/157.4.1819 PMC1461589

[40] Gilmour AR, Thompson R, Cullis BR (1995) Average information REML: An efficient algorithm for variance parameter estimation in linear mixed models. Biometrics 51: 1440–1450.

[41] Gilmour AR, Gogel BJ, Cullis BR, Thompson R (2009) ASReml User Guide Release 3.0. VSN International Ltd, Hemel Hempstead, HP1 1ES, UK www.vsni.c.

[42] R Development Core Team (2011) R: A language and environment for statistical computing.

[43] Cooper GM, Stone EA, Asimenos G, Green ED, Batzoglou S, et al (2005) Distribution and intensity of constraint in mammalian genomic sequence. Genome research 15: 901–913. 15965027 10.1101/gr.3577405 PMC1172034

[44] Flicek P, Ahmed I, Amode MR, Barrell D, Beal K, et al (2013) Ensembl 2013. Nucleic acids research 41: D48–55. 23203987 10.1093/nar/gks1236 PMC3531136

[45] Larkin MA, Blackshields G, Brown NP, Chenna R, McGettigan PA, et al (2007) Clustal W and Clustal X version 2.0. Bioinformatics (Oxford, England) 23: 2947–2948. 10.1093/bioinformatics/btm404 17846036

