# Supplementary material for: Expression Variants of the Lipogenic AGPAT6 Gene Affect Diverse Milk Composition Phenotypes in Bos taurus
Source: PLoS One. 2014 Jan 21;9(1):e85757. doi: 10.1371/journal.pone.0085757 (PMC3897493; doi:10.1371/journal.pone.0085757)
Supplement: Methods S1 — Supplementary methods. (DOCX) [file pone.0085757.s008.docx]

**Methods S1**

**Milk Composition Analysis**

For animals in the MA population (32,530 animals), milk composition records were restricted to first lactation measurements from October to January inclusive (spanning 86-172 days post-partum) and were required to meet the following criteria: milk volume > 5 litres, milk somatic cells <250k, and days of lactation > 30 days postpartum. Additionally, any records which were more than 5 standard deviations from the mean were excluded from the dataset. Animals were only included for analysis if they had at least two herd-test records that met all of the above criteria, resulting in 32,530 animals with milk fat percentage measurements, 32,526 animals with milk fat yield measurements, 32,505 animals with milk protein percentage measurements, 32,528 animals with milk protein yield measurements, and 32,526 animals with milk volume measurements. The slight discrepancies between these numbers reflect the removal of animals based on outlier criteria. Milk lactose percentage and yield measurements were available on a smaller subset of animals, consisting of 19,519 and 19,635 animals respectively. For FJX F2 cows (711 animals), higher resolution milk testing data was available, with six to seven composite am/pm samples taken over a three month period in mid-lactation (spanning 86-172 days post-partum) in the animals’ second lactation.

For fatty-acid analysis of milk fat in the FJX animals, composite am/pm milk samples were taken at peak (35 days post-partum), mid (87-104 days post-partum) and late lactation (198-206 days post-partum) in the animals’ second lactation. Milk samples were centrifuged at 1500g  for 10 minutes, and cream was removed from the sample and stored at -80°C until extraction  by a modification of the Röse Gottlieb technique [1]. Fatty acid methyl esters were formed by methoxide catalysed methylation [2] and fatty acid profiles quantified by gas-liquid chromatography. Fatty-acid methyl ester solutions were diluted to an appropriate concentration before on-column injection. Gas chromatographic analyses were performed on a Shimadzu GC17A (Shimadzu Corporation) equipped with a 30-m AT-1000 column (30 m × 0.32 mm i.d. and 0.25 ìm film thickness; Alltech Associates, Inc., Deerfield, IL) with hydrogen as the carrier gas (32 kPa). The flame ionization detector was set at 270°C. The column was operated at an initial temperature of 50°C for 1.5 min, then temperature programmed at 15°C/min to 150°C followed by 6°C/min to 220°C, where it was held for 26.2 min. On-column injection (0.2 uL) was used, with an initial temperature of 80°C before ramping to 150°C at 250°C/min, and then 220°C at 150°C/min. The relative proportions of individual fatty acids were calculated as grams per 100g of total fatty acid, and used for association analysis. For simplification of presentation, groups of individual fatty acids were also combined into categories and used for association analysis. These groupings consisted of the following: C4-C15: sum of all fatty acids C4 to C15. C16: sum of C16:0, C16:0 Br and C16:1. C17-C24: sum of all fatty acids C17 to C24. Omega-3: sum of C18:3n3, C18:4n3, C20:3n3, C20:4n3, C20:5n3, C22:5n3, C22:6n3. Omega-6: sum of C18:2n6, C20:2n6, C20:3n6, C20:4n6, C22:4n6, C22:5n6. Saturated: sum of all saturated fatty acids C4 to C24 (including branched). Unsaturated: sum of all unsaturated fatty acids C10:1 to C24. Poly-unsaturated: sum of ½ unknown peak A, C18:2n6, unknown peak B, C18:3n3, C18:2conjc9t11, C20:2n6, C20:3n6, C20:4n6, C20:3n3, C20:4n3, C20:5n3, C22:4n6. C22:5n6, C22:5n3, C22:6n3. Branched: sum of C13:0 Br, C14:0 Br, C15:0 isoBr, C15:0 anteisoBr, C16:0 Br, C17:0 isoBr, C17:0 anteisoBr. Cis-monoenoic: sum of C10:1, C12:1, C14:1, C16:1, C17:1, ½ unknown peak A, C18:1n9, C20:1n11, C20:1n9, C22:1n13, C22:1n9, C24:1n9. Trans-11-monoenoic: C18:1n7 (vaccenic). Unknown peak A was identified as a mixture of PUFA and cis-monoenoic from the chromatographic retention time and subsequent thin-layer chromatography. Unknown peak B was assumed as PUFA as indicated by the same methods.

**Genomic DNA extraction and ARS-BFGL-NGS-57448 SNP genotyping**

In the FJX population, genomic DNA was prepared as described previously [3], for the MA population, genomic DNA was extracted by two different methods. The first method used ear-punch tissue samples with Qiagen BioSprint kits (Qiagen) in conjunction with KingFisher Flex robotics (Thermofisher), and was conducted by GeneMark (Hamilton, New Zealand). The second method used ear-punch tissue samples processed using a MagMAX system (Life Technologies), with extractions performed by GeneSeek (Lincoln, NE, USA). For the 188 animals that were biopsied for RNA-seq in 2013, DNA extraction was also performed by GeneSeek, using the MagMAX system. For the 12 non-FJX animals that were analysed by qPCR, genomic DNA was extracted from mammary tissue using the Qiagen DNeasy Blood & Tissue kit and protocol (Qiagen). The Illumina BovineHD BeadChip SNP ARS-BFGL-NGS-57448 was manually genotyped on these 12 samples using PCR and Sanger sequencing with the following primers: ARS_BFGL_NGS_57448_For ATCTTTGCCAGCTCCCTGTC, ARS_BFGL_NGS_57448_Rev GCAAGAATCCATGCCTGCTG. Amplification was conducted in 10ul reactions using 0.4U of KAPA2G Robust DNA Polymerase and KAPA2G GC buffer (Kapa Biosystems), 400nM of each primer, 200uM dNTPs, and 20ng of gDNA template. Cycling conditions were: 95°C for 3 min; and 35 cycles of 95°C for 30 sec, 64°C for 30 sec, 72°C for 5 sec. This 221bp product was sequenced with the reverse PCR primer using Applied Biosystems BigDye version 3.1 terminator chemistry (Life Technologies/Applied Biosystems) on the Applied Biosystems 3130xL instrument (Life Technologies/Applied Biosystems) at the University of Auckland DNA Sequencing Facility (Auckland, New Zealand).

**RNA-seq - RNA extraction**

Total RNA was extracted by NZ Genomics Limited (NZGL; Auckland, New Zealand) using ~30mg of tissue added to 750ul of TRIzol Reagent (Life Technologies). Samples were homogenized using a PRO200 Homogenizer (PRO Scientific), and RNA extracted using QIAGEN RNeasy Columns (QIAGEN). Integrity of total RNA was assessed using an Agilent Bioanalyzer 2100 (Agilent) in conjunction with the RNA 6000 LabChip kits (Agilent). Prior to sequencing, total RNA was quantified using the Qubit instrument (Life Technologies).

**RNA-seq mapping and bioinformatics**

Average sequence yields were 93 million and 102 million high quality 100-bp paired-end reads for the 29 FJX and 188 Holstein-Friesian samples respectively. Sequence data were mapped to the UMD 3.1 genome using Tophat2 (version 2.0.8; 26) with a maximum mismatch parameter of four. Mapping located an average of 87 million read-pairs for the 29 FJX animals, and 84 million for the 188 Holstein-Friesian animals. Cufflinks software (version 2.1.1) [4] was used to quantify expressed transcripts, using options for no effective length correction, maximum bundle fragments of two million, and minimum intron lengths of 20bp. Genes with very high expression (such as the casein cluster of genes on chromosome 6) were masked out for this analysis. The transcripts assembled by Cufflinks were then merged with the Ensembl UMD3.1 GTF annotation (revision 70) using Cuffmerge (version 2.1.1) [4]. Tophat2 was then re-run using the merged GTF file as a reference set of gene model annotations. Cufflinks was then re-run using these Tophat2 results. This yielded fragments per kilobase of exon model per million mapped (FPKM) expression values for the 10 genes in the chromosome 27 1Mbp region of interest. The RNA-seq read-depth graph (Figure 2C) was generated using the coverage tool from the BEDTools suite of software (version 2.17.0) [5], by calculating the read depth at each base position for all Ensembl genes (revision 70). From these files, the median values were calculated over a subset of 97 animals using the merge tool from BEDTools, and used to graph read depth in the 1Mbp region of interest.

***AGPAT6* quantitative RT-PCR**

All RNA samples were treated with DNase I as previously described [6]. For cDNA synthesis, 750ng of total RNA was reverse transcribed using random hexamer primers and the Invitrogen Superscript III Supermix kit (Invitrogen), following the manufacturer’s guidelines. Reverse transcription negative control samples were generated by processing duplicate experimental samples as described above with the omission of the Superscript III enzyme. Templates were diluted 1/10 in water prior to PCR amplification on the Roche Lightcycler 480 instrument (Roche Diagnostics). A custom intron-spanning assay targeting exons 7 & 8 of the *AGPAT6* transcript reference sequence (NM_001083669.1) was designed using Roche Universal Probe Library (UPL) software (Roche). This assay used UPL probe #49, and the following forward and reverse primers: *AGPAT6*_For GCTCCGAAGTGAAGGATCG; *AGPAT6*_Rev GCTTTTATCCTGCACATGCTC. Two additional assays targeting *EIF3K* and *RPS15A* genes were designed to serve as endogenous controls for PCR normalisation. Primers and UPL probes for these assays were: *EIF3K*_For AAGTTGCTCAAGGGGATCG, *EIF3K*_Rev TTGGCCTGTGTCTCCACATA, UPL probe #1; *RPS15A*_For TCAGCCCTAGATTTGATGTGC, *RPS15A*_Rev GCCAGCTGAGGTTGTCAGTA, UPL probe #32. Amplification was conducted using Lightcycler 480 Probes Master mix (Roche) in a 10ul reaction volume, using 200nM of each primer, 100nM of hydrolysis probe, and 2ul of diluted cDNA template. Cycling conditions were: 95°C for 10 min; 45 cycles of 95°C for 10 sec, 60°C for 30 sec; and 40°C for 30 sec. All experimental and standard curve samples were amplified in quadruplicate, which included reverse transcriptase negative controls and negative controls lacking template. Absolute quantification of individual transcript levels was performed using the Roche Lightcyler 480 software second derivative maximum method, incorporating a 5-point standard curve. Relative quantification was then performed by dividing mean *AGPAT6* concentration values by mean endogenous control gene values, yielding normalised ratios of *AGPAT6* transcript to each endogenous control gene for each sample. The geometric mean of these values was then normalised to a calibrator sample to transform data to positive values for downstream data presentation and statistics.

**Genome sequence informatics and variant calling**

Sequence data was mapped to the UMD3.1 *Bos taurus* genome build using RTG map v2.3 ([www.realtimegenomics.com](http://www.realtimegenomics.com)), using parameters optimised for use with bovine sequence. Mapping of these data yielded an average read depth of 51.7x, with a mean breadth of 99.6% per sample. Short indels (-9bp to +9bp), SNPs and MNPs were called against RTG-mapped data across the six F1 sires using RTG SNP with default parameters. The population variant caller FreeBayes (v0.8.9; <http://bioinformatics.bc.edu/marthlab/FreeBayes>), was also used to call variants using the RTG-mapped data. Genome sequence was also mapped with BWA 0.5.9 [7] following the human 1,000 genomes pipeline, and a further subset of variants called using the GATK Unified Genotyper [8], utilising the BWA assembly. A list representing the union of these data was then annotated with *in silico* functions using SNPeff (http://snpeff.sourceforge.net) [9], and used for downstream filtering.

**Filtering and custom genotyping of sequence-variants**

Seventy-three SNPs and three indel variants were submitted for Sequenom iPLEX design. This represented a subset of the total variants in the 40kb region, with variants removed due to likely false positive genotype assignment (following manual inspection of sequence alignments), and indel variants within polynucleotide tracts that could not be typed using the Sequenom platform (none of which mapped to high priority gene regions such as exons or splice sites*).* For pragmatic reasons, including genotype cost, a subset of variants within the large first intron and 5’ region of *AGPAT6* were also omitted from the target variant set. Sire variants that were deemed high priority due to being in strong linkage disequilibrium (LD; R^2^>0.75) with the top milk fat percentage-associated SNP ARS-BFGL-NGS-57448 were not subjected to this filter. A high priority indel variant chr27 g.36198118GGC(4_5) VNTR that could not be typed using the Sequenom platform was instead targeted using a fluorescent PCR-based assay. This assay used a FAM-labelled forward primer and unlabelled reverse primer to amplify a 176bp fragment encompassing this polymorphism. These primers were VNTR_For CAAGGCGGCGTAGACAAA, VNTR_Rev AGCCCCGCTAGAGGTTCAT. Templates were amplified in 10ul reaction volumes using 0.4U of KAPA2G Robust DNA Polymerase and KAPA2G GC buffer (Kapa Biosystems), 200nM of each primer, 200uM dNTPs, and 10ng of gDNA template. Cycling conditions were: 95°C for 3 min; and 35 cycles of 95°C for 30 sec, 60°C for 30 sec, 72°C for 30 sec. Fluorescently labelled PCR products were diluted 10-fold and 2ul of each sample was run on the Applied Biosystems 3130xL instrument (Life Technologies/Applied Biosystems), at the University of Auckland DNA Sequencing Facility (Auckland, New Zealand). The same 812 FJX F2 DNA samples were targeted for all custom assays. Table S7 details the filtering steps from raw variants derived from automated low stringency calling of genome sequence data, to manually curated target variants, to the quality-filtered variants used for association analysis.

**Quality filtering of custom genotypes**

Prior to association analysis, custom genotyping data were quality-filtered to remove individuals with less than 80% call rate across all SNPs (13 individuals), and variants with less than 80% call rate across all individuals (eight variants). Nine variants were excluded due to low minor allele frequency (<1%), necessarily representing false positive genotype calls from sequence data since variants were discovered in the six F1 sires that represent half the chromosomes in the F2 pedigree. Three variants were excluded on the basis of a non-conservative Hardy-Weinberg equilibrium threshold of P<1x10-6. These criteria yielded 59 variants (Table S6 & S7), with missing genotypes in the remaining individuals then imputed using Beagle software (v3.3.2) [10].

1. International Dairy Federation (1987) Milk. Determination of fat content – Rose Gottlieb gravimetric method.

2. MacGibbon AKH (1988) Modified method of fat extraction for solid fat content determination. New Zealand Journal Of Dairy Science & Technology 23: 399–403.

3. Berry SD, Lopez-Villalobos N, Beattie EM, Davis SR, Adams LF, et al. (2010) Mapping a quantitative trait locus for the concentration of beta-lactoglobulin in milk, and the effect of beta-lactoglobulin genetic variants on the composition of milk from Holstein-Friesian x Jersey crossbred cows. New Zealand veterinary journal 58: 1–5.

4. Trapnell C, Williams BA, Pertea G, Mortazavi A, Kwan G, et al. (2010) Transcript assembly and quantification by RNA-Seq reveals unannotated transcripts and isoform switching during cell differentiation. Nature biotechnology 28: 511–515.

5. Quinlan AR, Hall IM (2010) BEDTools: a flexible suite of utilities for comparing genomic features. Bioinformatics (Oxford, England) 26: 841–842.

6. Littlejohn MD, Walker CG, Ward HE, Lehnert KB, Snell RG, et al. (2010) Effects of reduced frequency of milk removal on gene expression in the bovine mammary gland. Physiological genomics 41: 21–32.

7. Li H, Durbin R (2010) Fast and accurate long-read alignment with Burrows-Wheeler transform. Bioinformatics (Oxford, England) 26: 589–595.

8. McKenna A, Hanna M, Banks E, Sivachenko A, Cibulskis K, et al. (2010) The Genome Analysis Toolkit: a MapReduce framework for analyzing next-generation DNA sequencing data. Genome research 20: 1297–1303.

9. Cingolani P, Platts A, Wang LL, Coon M, Nguyen T, et al. (n.d.) A program for annotating and predicting the effects of single nucleotide polymorphisms, SnpEff: SNPs in the genome of Drosophila melanogaster strain w1118; iso-2; iso-3. Fly 6: 80–92.

10. Browning BL, Browning SR (2009) A unified approach to genotype imputation and haplotype-phase inference for large data sets of trios and unrelated individuals. American journal of human genetics 84: 210–223.
